# Effects of Fermented Citrus Peel on Ameliorating Obesity in Rats Fed with High-Fat Diet

**DOI:** 10.3390/molecules27248966

**Published:** 2022-12-16

**Authors:** Chung-Hsiung Huang, Shun-Yuan Hsiao, Yung-Hsiang Lin, Guo-Jane Tsai

**Affiliations:** 1 Department of Food Science, National Taiwan Ocean University, Keelung 20224, Taiwan; 2 Research and Design Center, TCI Co., Ltd., Taipei 11494, Taiwan; 3 Center for Marine Bioscience and Biotechnology, National Taiwan Ocean University, Keelung 20224, Taiwan

**Keywords:** anti-obesity, fermented citrus peel, high-fat diet, hypolipidemic

## Abstract

Although citrus peel is a waste material, it contains a variety of bioactive components. As our preliminary findings showed that citrus peels fermented with *Saccharomyces cerevisiae* T1 contained increased levels of anti-obesity flavonoids, the objective of this study was to prepare fermented citrus peel and to investigate its effect on ameliorating obesity in Sprague Dawley (SD) rats fed with a high-fat diet (HFD). After fermentation, the amounts of limonene, nobiletin and 3-methoxynobiletin in citrus peel were markedly increased. SD rats were fed with an HFD for 10 weeks, followed by fermented citrus peel-containing HFD (0.3% or 0.9% *w*/*w*) for 6 weeks. Compared with those fed with an HFD alone, lower levels of body weight, visceral fat, body fat percentage, blood triglyceride, total cholesterol, low-density lipoprotein, malondialdehyde and hepatic adipose accumulation were observed in rats fed with fermented citrus peel. In parallel, hepatic levels of acetyl-CoA carboxylase and fatty acid synthase were diminished, and the level of hormone sensitivity lipase in visceral fat was elevated. These results reveal fermented citrus peel is a promising natural product with beneficial effects of alleviating HFD-induced obesity.

## 1. Introduction

In the past decades, obesity has become a public issue around the world, and the obesity prevalence has continuously increased. Obesity is a type of metabolic syndrome, which results in an abnormal increase in adipose tissue due to an imbalance in energy intake and expenditure. In particular, obesity is a crucial risk factor in eliciting or deteriorating cardiovascular disease, type 2 diabetes, fatty liver disease, etc. [1,2]. It has been reported that chronic high-fat diet (HFD) exposure leads to obesity, visceral fat accumulation and hyperlipidemia, which is characterized by a decrease in high density lipoprotein and an increase in low density lipoprotein and total cholesterol [3]. In addition, obesity-induced insulin resistance increases plasma triglyceride levels, which then lead to the accumulation of high levels of triglyceride and free fatty acids in liver and adipose tissue [4]. Due to increases in obese population and obesity-related complications, the development of natural products or functional foods for the management of obesity has attracted attention.

It has been reported that higher activity of acetyl-CoA carboxylase (ACC), which catalyzes the carboxylation of acetyl-CoA to malonyl-CoA, and fatty acid synthase (FAS), which catalyzes the synthesis of fatty acids from malonyl-CoA, was observed in obese individuals than in healthy ones [5]. Both ACC and FAS are key enzymes in the adipogenesis pathway, implying a causal relationship between the consequences of excess energy intake and the development of obesity [6]. On the other hand, hormone-sensitive lipase (HSL) and lipoprotein lipase (LPL) are known to catalyze the hydrolysis of triglyceride [7]. Suppression of HSL and LPL activity would diminish the level of triglyceride and elevate the level of triglyceride in plasma [8].

The global annual production of citrus fruits was 48.8 million tons from 2021 to 2022, and *Citrus reticulata* accounts for 70% of production. Citrus wastes such as pomace, orange peel and seeds are generated during processing, and peel accounts for 50% of the total dry weight of citrus [9]. The contents of crude fiber, crude protein, ash and crude fat in citrus peel are 57.0%, 10.2%, 3.33% and 2.22%, respectively. Citrus peel also contains 0.4–1.6% of essential oils and terpenes; limonene is the major one, and consists 90% of essential oils [10]. In addition, citrus peel is rich in various flavonoid derivatives, including nobiletin, and hydroxy polymethoxy flavones (PMFs) [11]. Although the anti-obesity potential of citrus-derived components has been reported, a novel process to increase the content of available bioactive compounds within citrus peel products is required [12]. In previous studies, hydrocarbon hydroxylases have been utilized to enhance the bioactive component content and bioactivity of citrus extracts. Recently, refinement and reuse of agricultural wastes by fermentation has attracted attention because of they may benefit environmental sustainability. For example, Wilkins et al. have processed lemon peel by enzymatic hydrolysis and fermentation with *Saccharomyces cerevisiae* to increase the amount of essential oil and ethanol obtained [13].

Chenpi has been obtained from the dry peel of *Citrus reticulate* using post-fermentation and maturation for many years. It has been used as traditional Chinese medicine for a long time to treat dyspepsia and airway inflammation. Recently, more and more studies have explored the influence of Chenpi on alleviating HFD-induced obesity, and modulation of gut microbiota is considered one of its action mechanisms [14,15,16,17]. Yang et al. showed that the storage time of Chenpi significantly affects its active ingredient content, and they identified a total of 47 different compounds, mainly flavonoids, in Chenpi [18]. These results indicated that post-fermentation and maturation processes are crucial for the quality and bioactivities of Chenpi.

In our preliminary study, fermentation of citrus peel with *S. cerevisiae* T1 significantly increased levels of limonene, nobiletin and 3-methoxynobiletin. Since the effects of limonene and nobiletin on modulating lipid metabolism have been reported [19,20,21,22], it was hypothesized that the fermented citrus peel may exert beneficial influence on ameliorating HFD-induced obesity. In this study, HFD-induced obese rats were orally treated with fermented citrus peel extract for 6 weeks, and the endpoints, including body fat, blood lipids, hepatic lipid accumulation and lipid peroxidation—which are key factors leading to HFD-induced metabolic syndrome—were measured to understand the anti-obesity potential of fermented citrus peel.

## 2. Results

### 2.1. Changes in the Contents of Limonene, Nobiletin and 3-Methoxynobiletin within Fermented Citrus Peel

**Figure 1:**
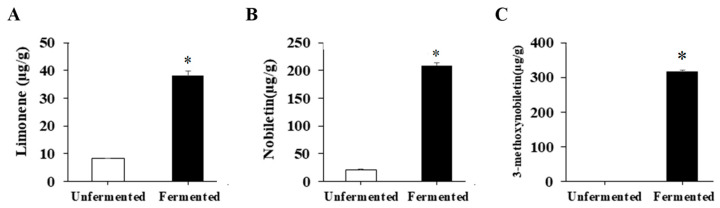
The levels of limonene, nobiletin and 3-methoxynobiletin in unfermented and fermented citrus peel. The citrus peel was fermented, and the levels of (**A**) limonene, (**B**) nobiletin and (**C**) 3-methoxynobiletin in unfermented and fermented citrus peel were determined by HPLC as described in the Materials and Methods section. Result are expressed as mean ± SD (*n* = 3). * *p* < 0.05 compared with unfermented citrus peel.

Because limonene, nobiletin and 3-methoxynobiletin are well-known anti-obesity components within citrus peel, the amounts of these components were investigated. Compared with that without fermentation, the fermented citrus peel contained markedly higher levels of these components (Figure 1), revealing that fermentation of citrus peels by *S. cerevisiae* T1 is a promising process to raise limonene, nobiletin and 3-methoxynobiletin levels.

### 2.2. Effect of Fermented Citrus Peel on Ameliorating Obesity in Rats Fed with HFD

**Figure 2:**
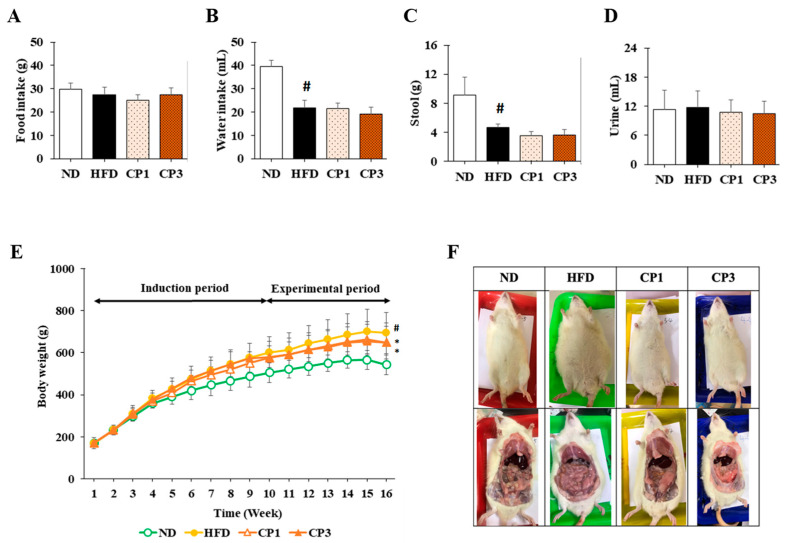
Week-average of (**A**) feed intake, (**B**) water intake, (**C**) stool weight, (**D**) urine volume, (**E**) body weight, and (**F**) representative photos of appearance and abdominal organ of rats. The rats in each group were treated as described in the Materials and Methods section, and the feed intake, water intake, stool weight, urine volume, and body weight of each rat were measured and recorded weekly. After 16-week treatment, the rats were sacrificed, and the photos of appearance and abdominal organs of rats were taken individually. Results are expressed as mean ± S.D. for each group of rats (*n* = 6). # *p* < 0.05 compared with the ND group; * *p* < 0.05 compared with the HFD group.

**Figure 3:**
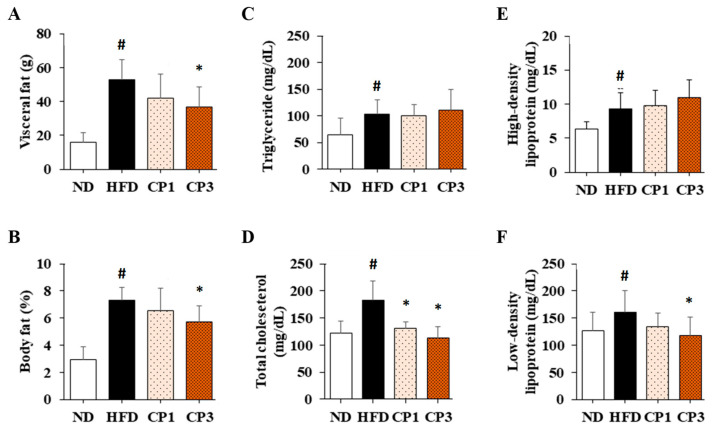
The average (**A**) visceral adipose weight, (**B**) body fat percentage and serum levels of (**C**) triglyceride, (**D**) total cholesterol, (**E**) high-density lipoprotein and (**F**) low-density lipoprotein of rats. After 16-week treatment, the rats were sacrificed, and the visceral fat was isolated and weighed to calculate body fat percentage. Moreover, the blood samples were harvested to measure serum levels of triglyceride, total cholesterol, high-density lipoprotein and low-density lipoprotein as described in the Materials and Methods section. Results are expressed as mean ± S.D. for each group of rats (*n* = 6). # *p* < 0.05 compared with the ND group; * *p* < 0.05 compared with the HFD group.

**Figure 4:**
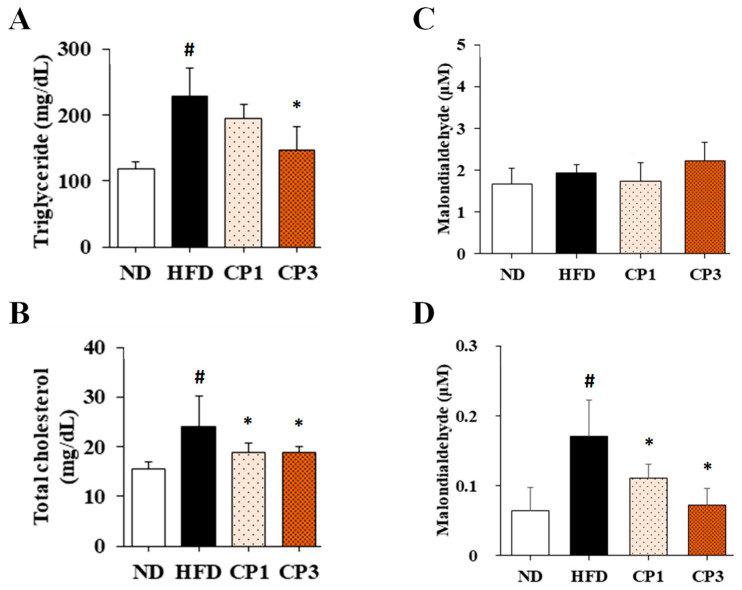
The levels of (**A**) triglyceride, (**B**) total cholesterol, (**C**) malondialdehyde in liver and (**D**) malondialdehyde in serum of rats. After 16-week treatment, the rats were sacrificed, and the liver and blood samples were harvested to measure the levels of triglyceride, total cholesterol and malondialdehyde as described in the Materials and Methods section. Results are expressed as mean ± S.D. for each group of rats (*n* = 6). # *p* < 0.05 compared with the ND group; * *p* < 0.05 compared with the HFD group.

**Figure 5:**
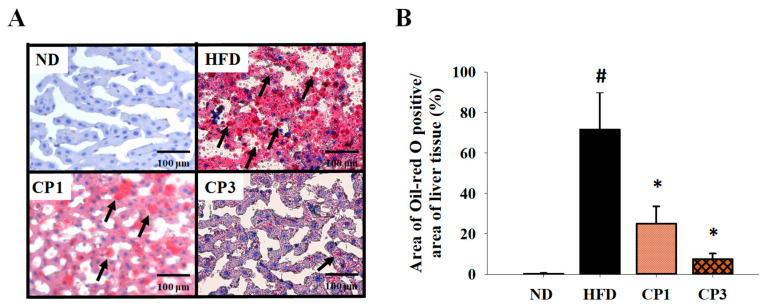
Histopathological analysis of liver tissue stained with oil red O. After 16-week treatment, the rats were sacrificed, and the liver samples were isolated to prepare paraffin-embedded sections for oil red O staining. (**A**) Representative photos are shown. Scale bar = 100 μm. Arrows indicate lipid droplets. (**B**) The percentage of oil red O positive area in the area of liver tissue was calculated by ImageJ image processing and analysis program. Results are expressed as mean ± S.D. for each group of rats (*n* = 6). # *p* < 0.05 compared with the ND group; * *p* < 0.05 compared with the HFD group.

To understand the anti-obesity activity of fermented citrus peel, the rats fed with HFD for 10 weeks were subsequently fed with a fermented citrus peel-containing HFD (0.3% or 0.9% *w*/*w*; CP1 or CP3) for 6 weeks. Compared with those fed with an HFD alone, comparable levels of average food intake, water intake, stool weight and urine volume were observed in rats of CP1 and CP3 groups (Figure 2A–D). However, the body weight of rats in CP1 and CP3 groups was lower than that of those in the HFD group (Figure 2E). On the day of sacrifice, the body weight of rats in the ND, HFD, CP1 and CP3 groups were 542.66 ± 45.50 g, 693.47 ± 98.20 g, 648.29 ± 77.04 g and 648.01 ± 91.97 g, respectively. Moreover, rats in the CP1 and CP3 groups showed a slimmer appearance and abdominal organs compared with those in the HFD group (Figure 2F). In parallel, fermented citrus peel treatment alleviated visceral adipose weight (from 52.76 ± 11.78 g to 36.54 ± 12.00 g), body fat percentage (from 7.32 ± 0.93% to 5.71 ± 1.20%), serum total cholesterol levels (from 182.64 ± 34.88 mg/dL to 112.50 ± 21.91 mg/dL) and low-density lipoprotein (from 161.21 ± 39.44 mg/dL to 117.55 ± 33.54 mg/dL) in a dose-dependent manner, although triglyceride and high-density lipoprotein levels were not significantly influenced by fermented citrus peel treatment (Figure 3). Since a fat-rich diet can induce hepatic lipid accumulation and lipid peroxidation [23], the hepatic levels of triglyceride, total cholesterol and malondialdehyde and serum level of malondialdehyde were measured. Chronic HFD treatment significantly elevated the hepatic levels of triglyceride (from 117.98 ± 11.79 mg/dL to 227.58 ± 43.57 mg/dL), total cholesterol (from 15.55 ± 1.28 mg/dL to 24.15 ± 5.97 mg/dL) and serum level of malondialdehyde (from 0.06 ± 0.03 μM to 0.17 ± 0.05 μM) (Figure 4). Fermented citrus peel treatment effectively reduced hepatic levels of triglyceride (from 227.58 ± 43.57 mg/dL to 146.38 ± 35.42 mg/dL), total cholesterol (from 24.15 ± 5.97 mg/dL to 18.85 ± 1.08 mg/dL) and serum level of malondialdehyde levels (from 0.17 ± 0.05 μM to 0.07 ± 0.02 μM) (Figure 4). Hepatic MDA levels were comparable between all groups conceivably due to the richness of antioxidant enzymes in the liver. These results reveal that fermented citrus peel has the potential to improve HFD-induced hepatic lipid accumulation and lipid peroxidation. Further, the in situ effects of fermented citrus peel on HFD-induced hepatic lipid accumulation were explored by histopathological examination of oil red O-stained liver tissue sections. Significant lipid droplets were observed in the liver of the HFD-fed rats compared with the ND rats (Figure 5A). Notwithstanding, fewer and limited numbers of lipid droplets were observed in rats of the CP1 and CP3 group, respectively (Figure 5A). Through image processing and analysis, fermented citrus peel treatment was shown to reduce the percentage of oil red O-positive area in the liver tissue, in a dose-dependent manner (from 71.67 ± 18.35% to 7.5 ± 2.74%), indicating the beneficial effect of fermented citrus peel on attenuating hepatic lipid accumulation (Figure 5B).

### 2.3. Effect of Fermented Citrus Peel on Modulating Activities of Lipid Metabolic Enzymes in Rats Fed with HFD

**Figure 6:**
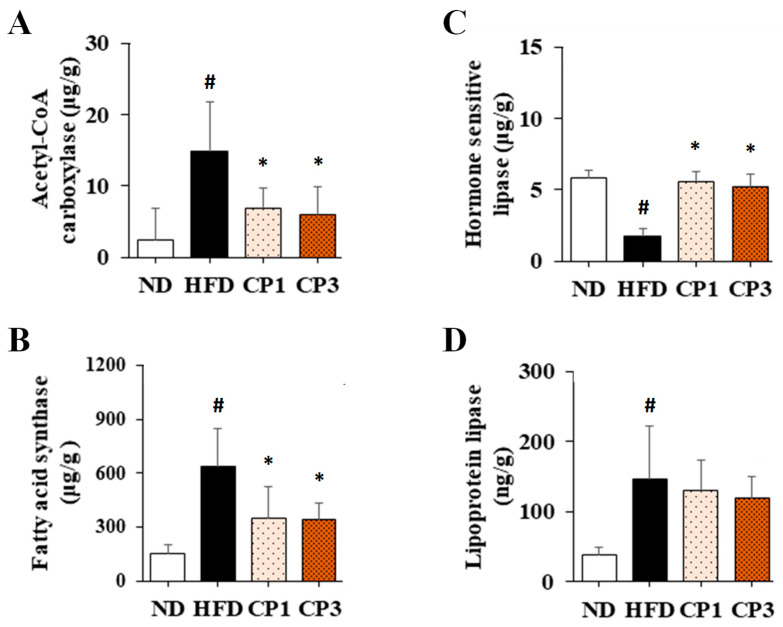
The levels of (**A**) acetyl-CoA carboxylase and (**B**) fatty acid synthase in liver and (**C**) hormone sensitivity lipase and (**D**) lipoprotein lipase in visceral fat of rats. After 16-week treatment, the rats were sacrificed, and the liver and visceral fat samples were isolated to determine the levels of acetyl-CoA carboxylase, fatty acid synthase, hormone sensitivity lipase and lipoprotein lipase, respectively, as described in the Materials and Methods section. Results are expressed as mean ± S.D. for each group of rats (*n* = 6). # *p* < 0.05 compared with the ND group; * *p* < 0.05 compared with the HFD group.

To further evaluate the potential mechanism of fermented citrus peel-mediated anti-obesity effect, the levels of acetyl-CoA carboxylase (ACC) and fatty acid synthase (FAS) in liver and hormone sensitivity lipase (HSL) and lipoprotein lipase (LPL) in visceral fat were illustrated. Chronic HFD exposure significantly raised the hepatic levels of ACC (from 2.42 ± 4.46 μg/g to 14.78 ± 7.02 μg/g) and FAS (from 151.95 ± 49.40 μg/g to 640.54 ± 210.0 μg/g), whereas fermented citrus peel treatment obviously declined HFD-induced increment of ACC (from 14.78 ± 7.02 μg/g to 6.00 ± 3.85 μg/g) and FAS levels (from 640.54 ± 210.0 μg/g to 344.51 ± 88.03 μg/g) (Figure 6A,B). On the other hand, chronic HFD exposure reduced the HSL level (from 5.81 ± 0.54 μg/g to 1.74 ± 0.53 μg/g) (Figure 6C). However, fermented citrus peel treatment reversed the diminished HSL level (from 1.74 ± 0.53 μg/g to 5.55 ± 0.74 μg/g) (Figure 6C). Interestingly, HFD enhanced the LPL level (from 38.12 ± 10.19 ng/g to 146.61 ± 74.62 ng/g), and fermented citrus peel had limited impact on HFD-induced LPL increment (from 146.61 ± 74.62 ng/g to 118.03 ± 30.92 ng/g) (Figure 6D).

## 3. Discussion

In the current study, a biorefinery process of citrus peel waste fermented with unique *S. cerevisiae* T1 was established. The significantly increased contents of limonene, nobiletin, and 3-methoxyligenin provide a promising application of fermented citrus peels for the treatment of HFD-induced obesity, which was evidenced by the results of animal experiments.

The beneficial effects of various varieties of citrus peel on improving obesity and metabolic disorders have been demonstrated from in vitro experiments to in vivo experiments and even clinical trials. In 3T3-L1 adipocytes, the citrus (*C. unshiu*) peel extract (500 μg/mL) inhibited lipid and triglyceride accumulation and suppressed glycerol-3-phosphate dehydrogenase perilipin mRNA expression [24]. The obese and diabetic mice treated daily with citrus (*C. grandis*) peel extract (1.2 g/kg) for 3 weeks showed weight loss. The anti-obesity and anti-diabetic effects of citrus peel extract were associated with increased lipase and hexokinase activities and decreased glucose 6-phosphatase activity [25]. In addition, HFD-fed mice were orally administered ethanolic extracts of oranges, lemons, limes, tangerines, grapefruit peels or synergistic combinations in equal proportions (500 mg/kg/day for 14 days), and their body and liver weights as well as serum total cholesterol and triglyceride were decreased [26]. Supplementation with citrus peel extract containing 0.5% PMFs and hydroxyl PMFs for 16 weeks reduced adipose tissue weight and body weight in HFD-fed mice [12]. Citrus (*C. unshiu*) peel treatment (300 mg/kg/day for 12 weeks) reduced body weight gain gain, decreased epididymal fat, mesenteric fat, plasma and hepatic triglyceride levels and increased high-density lipoprotein. Fat accumulation in adipose tissue was decreased because of upregulation of lipolysis enzyme HSL, and downregulation of adipogenesis related genes such as C/EBPα and ACC [14]. Moreover, oral administration of Chenpi extract-containing food (0.25% and 0.5% *w*/*w*) for 15 weeks significantly prevented HFD-induced obesity, hepatic steatosis and diabetes in mice [27]. Recently, Wu et al. investigated the effects of fermented *Citrus limon* products on liver lipid metabolism and gut microbiota in a rat model of obesity caused by a high-calorie diet. The fermented products effectively reduced total triglyceride and total cholesterol contents in the liver. Additionally, the mRNA levels of genes related to triglyceride metabolism and lipid β-oxidation were regulated. Interestingly, a decreased ratio of *Firmicutes/Bacteroidetes* and an increased ratio of *Firmicutes/Clostridia* were observed, revealing the anti-obesity effect of fermented *Citrus limon* products occurs through modulating the lipid metabolism and improving the gut microbiota [28]. In clinical trials, the results of a randomized and double-blind pilot study show that oral treatment with citrus (*C. sudachi*) peel powder (1.3 g/day for 12 weeks) decreased body weight, waist circumference and serum triglyceride levels in participants with serum level of triglycerides greater than 120 mg/dL [29]. Laboratory and biometric readings before and after administration of citrus (*C. unshiu*) peel pellets (18 mg/day) for 4 weeks were analyzed in 118 patients with a body mass index (BMI) over 23 at a public health center. A significant decrease in BMI was observed in all treated subjects, with 65.3% of subjects losing 1.03 ± 0.83 kg and reducing total cholesterol, triglyceride and low-density lipoprotein levels after 4 weeks of treatment [30]. In another double-blind and placebo-controlled study, daily intake of 800 mg citrus (*C. reticulate*) peel dry extract for 8 weeks reduced BMI, body fat percentage, waist circumference and blood levels of total cholesterol and triglyceride in obese adolescent subjects [31].

Some reports also demonstrated the anti-obesity effects of unripe citrus peel. Unripened citrus (*C. sunki*) peel extract at the concentration of 400 μg/mL enhanced lipolysis in differentiated 3T3-L1 adipocytes by phosphorylating cAMP-dependent protein kinase and HSL [32]. Kang et al. demonstrated that unripened citrus (*C. sunki*) peel extract supplement (150 mg/kg/day for 70 days) reduced body weight gain, adipose tissue weight, serum levels of total cholesterol and triglyceride and the accumulation of fatty droplets in the liver of mice fed with an HFD. It also reversed HFD-induced reduction of AMP-activated protein kinase and ACC phosphorylation levels in epididymal adipose tissue which was associated with fatty acid beta oxidation [32]. Moreover, 5% unripened citrus (*C. aurantium*) peel supplementation for 4 weeks suppressed body weight gain and decreased epididymal, perirenal and subcutaneous fat weights, and blood levels of total cholesterol and triglyceride in HFD-fed mice [33].

Fermentation is an effective way to treat agricultural waste and to produce valuable products from organic bioresources. Phenolics present in plants are classified into free phenolics and bound phenolics. Free phenolics are easily extracted by conventional techniques. However, several hydrolysis processes are required to release bound phenolics. Fermentation is one of the best processes to release bound phenolics because the enzymes produced from the microorganism are capable of completely breaking down the bonds between phenolics and the cell-wall matrix. Additionally, numerous metabolic pathways of phenolics have been discovered by microbial fermentation [34]. Therefore, it is suggested that the increased amounts of limonene, nobiletin and 3-methoxynobiletin in fermented citrus peel may be due to the improved release and metabolism of flavonoids. In this study, citrus peel was fermented with *S. cerevisiae* T1, and the contents of limonene, nobiletin and 3-methoxynobiletin increased from 8.2, 20.9 and 0 μg/g to 38.0, 209.2 and 316.0 μg/g, respectively. Kimoto-Nira et al. fermented citrus peel with *Lactobacillus plantarum*, wherein the content of flavonoids was not altered [35]. It has been reported that the essential oils rich in citrus peel suppress the metabolic function of microorganisms, inhibit the growth of fermentation strains such as *Bacillus subtilis*, *S. cerevisiae*, *Aspergillus awamori*, and have toxicity to yeast [36,37,38]. Therefore, it is necessary to remove essential oils before fermentation. Wilkins et al. combined enzymatic hydrolysis and *S. cerevisiae* fermentation to treat lemon peel for 24 h, and 0.14% (*v*/*v*) lemon essential oil and 0.33% (*v*/*v*) alcohol were generated [13]. Tian et al. demonstrated that alcohol produced from *S. cerevisiae* fermentation raised the content of flavonoids, because alcohol could increase the solubility of flavonoids [39].

Limonene is a major component in citrus essential oils. In HFD-fed mice, limonene supplement (0.5% *w*/*w*) had preventive and therapeutic effects on decreasing the size of white and brown adipocytes, lowering serum levels of triglyceride, low-density lipoprotein and fasting blood glucose, and on improving glucose tolerance, increasing the serum level of high-density lipoprotein and preventing liver lipid accumulations [19]. A recent study showed that limonene treatment (30 and 60 mg/kg body weight for 84 days) decreased body weight gain in HFD-fed mice [20]. Nobiletin is a polymethoxylated flavone contained in citrus fruits. In HFD-fed mice, nobiletin treatment (10 or 100 mg/kg) decreased body weight gaine, weight of white adipose tissue and plasma levels of triglyceride and glucose, and improved plasma level of adiponectin and glucose tolerance, and altered the expression of lipid metabolism-related and adipokine genes [22].

So far, limited studies investigating the anti-obesity activity of fermented citrus peel are available. Jeon et al. indicated that extract of citrus peel with bio-transformed *Aspergillus oryzae* had stronger anti-obesity activity than unfermented citrus peel [40]. Recently, Pan et al. demonstrated that the lemon peel fermentation supernatant (LPFS) could inhibit weight gain in mice and improve the lesions of liver and epididymal adipose tissue. In addition, LPFS regulated blood lipids, liver function and inflammation-related indicators in the serum of obese mice [41]. 

In the current study, 0.3% and 0.9% fermented citrus peel intervention in rats indicates that the doses of fermented citrus peel were approximately 150 and 450 mg/kg body weight, which were lower or comparable to that reported in the previous animal studies and clinical trials after dose conversion [25,26,27,28,29,30,31,42]. Based on the results shown in Figure 1, the doses of limonene, nobiletin and 3-methoxynobiletin were approximately 6, 30 and 45 μg/kg body weight, respectively, which were far lower than that employed in previous studies [19,20,21,22]. Therefore, further study will be required to identify other anti-obesity compounds in fermented citrus peel and to clarify the synergistic potential of these compounds against obesity. The anti-obesity property of fermented citrus peel was evidenced by reduced levels of body weight, visceral fat, body fat percentage, blood triglyceride, total cholesterol, low-density lipoprotein, malondialdehyde and hepatic adipose accumulation in HFD-induced obese rats. Based on the results of average food intake, water intake, stool weight and urine volume, the anti-obesity activity of fermented citrus peel was not caused by changes in appetite and excretion. Similar findings from the previous studies showed that mice treated with Chenpi extract had reduced body weight gain and the same amount of food intake and feces excretion [27]. It is suggested that nutrient absorption rates were similar between CP3 and the HFD, and the lower body weight gain may be due to the suppression of fat accumulation rather than malnutrition. The action mechanism of fermented citrus peel was closely related to the modulation of lipid metabolism-related enzymes, such as ACC, FAS and HSL. It is consistent with the evidence reported by previous studies [14,19,21,24,32]. How fermented citrus peel modulates these enzymes is an interesting issue. Furthermore, Wu et al. showed that improvement of gut microbiota is a potential mechanism for the anti-obesity effects of citrus fermented products [28]. Therefore, further studies can be conducted by elucidating the cellular signaling pathways involved in the expression of these enzymes and the profile of gut microbiota. 

## 4. Materials and Methods

### 4.1. Chemicals and Reagents

All chemicals and reagents were purchased from Sigma-Aldrich Chemical Co. (St. Louis, MO, USA) unless otherwise stated. The rat cholesterol enzymatic kit and triglyceride enzymatic kit were purchased from Randox Laboratories, Ltd. (Crumlin, UK). Based on the experimental design of the previous study [43], the rodent normal diet D5001 and high fat diet D12451 were purchased from Laboratory Rodent Diet (LabDiet, St. Louis, MO, USA) and Research Diets, Inc. (New Brunswick, NJ, USA) and used for ND and HFD, respectively. Both D5001 and D12451 feeds contain 24% protein. Feed D5001 contains 5% fat and provides 13% of calories in a 4.09 Kcal/g feed; while Feed D12451 contains 24% fat and provides 45% of calories in a 4.73 Kcal/g feed. Feed D12451 was added to fermented citrus peel powder and mixed well with a blender to prepare the fermented citrus peel-containing HFD (0.3% or 0.9% *w*/*w*).

### 4.2. Preparation of Fermented Citrus Peel Powder Sample

The fermented citrus peel powder was prepared and provided by Research & Design Center of TCI CO., Ltd. (Taipei, Taiwan). Briefly, 50 g of citrus powder (*Citrus reticulate*) peel (CP) were added with 4540 mL deionized water and sterilized at 95 °C for 30 min. Then, the CP suspension was inoculated with the culture of *Saccharomyces cerevisiae* T1, which was activated in potato dextrose broth at 30 °C for 2 days, with an initial cell density of 1 × 10^6^ CFU/mL. After 5 days of fermentation at 30 °C, the culture filtrate was collected by filtration, pasteurized at 75 °C for 30 min and spray-dried. Finally, the obtained dry powder was mixed with an equal amount of maltodextrin to obtain the fermented CP powder sample.

### 4.3. Determination of Limonene, Nobiletin and 3-Methoxynobiletin

The amount of limonene, nobiletin and 3-methoxynobiletin in fermented citrus peel was determined by high performance liquid chromatography (HPLC; Hitachi chromaster 5260 series, Hitachi, Tokyo, Japan) with Mightysil RP-18 GP 250 analytical column (250 × 4.6 mm, 5 μm, Kanto, Tokyo, Japan) and Diode Array Detector (Hitachi chromaster 5430). Gradient elution was used with a mobile phase composed of methanol (solvent A) and water containing 0.1% formic acid (solvent B). The elution condition was as follows: the initial 3 min was started with A:B in a ratio of 10:90, followed by linearly increasing to the ratio of 100:0 at 25 min. The flow rate was 1 mL/min, and the column temperature was 40 °C. The detection wavelength was 254 nm, and the injection volume was 10 μL. Standard or sample solutions prepared by dissolving standards or samples in methanol and sonicated for 10 min were filtered with 0.22 mm polyvinylidene fluoride prior to injection. The quantitative standard curve was constructed to determine the levels of limonene, nobiletin and 3-methoxynobiletin.

### 4.4. Animals and Experimental Design

Five-week-old male Sprague Dawley rats were obtained from BioLASCO Taiwan Co., Ltd. (Taipei, Taiwan). On arrival, rats were housed in stainless steel cages with diet and water provided ad libitum for 1 week before treatment. Rats were kept under humid conditions (30–70%) and 12 h light/dark cycle in a temperature (23 °C ± 2 °C)-controlled environment. All animal experiments were performed in accordance with the guidelines for the care and use of laboratory animals and approved by the Institutional Animal Care and Use Committee of the National Taiwan Ocean University (NTOU-110026). The rats were randomly divided into 4 groups (*n* = 6). Except for rats fed with normal diet (ND), the other rats were fed with high-fat diet (HFD) for 10 weeks followed by HFD alone, or HFD containing fermented citrus peel (0.3% or 0.9% *w*/*w*; CP1 or CP3) for another 6 weeks. Food and water intake, body weight, urine output, and stool weight were measured weekly. After sacrifice, the visceral fat and liver tissues were collected for further analysis.

### 4.5. Measurement of Triglyceride, Total Cholesterol and Lipoprotein–Cholesterol Levels in the Plasma

Blood samples were collected at sacrifice for the isolation of plasma by centrifugation. Plasma concentrations of triglyceride and total cholesterol were measured using enzymatic kits (Randox Laboratories Limited, Crumlin, UK) according to the manufacturer’s instructions. A modified phosphotungstic acid/magnesium chloride precipitation procedure was used to isolate low-density lipoprotein and high-density lipoprotein [44]. The concentrations of cholesterol in the high-density lipoprotein-containing supernatants and low-density lipoprotein-containing precipitations were measured using enzymatic kits (Randox Laboratories Limited, Crumlin, UK) according to the manufacturer’s instructions. The quantitative standard curve was constructed to determine triglyceride, total cholesterol and lipoprotein–cholesterol levels.

### 4.6. Measurement of Triglyceride and Total Cholesterol Levels in the Liver Tissues

The liver tissues were mixed with chloroform/methanol solution and homogenized (SA-50 Max. 3000; Hong Sheng, Taipei, Taiwan) for 1 min. Supernatants were harvested by centrifugation for lipid acquisition and mixed with Triton X-100, followed by solvent removal using vacuum evaporation (SC 110; Savant Instruments, Holbrook, NY, USA) [45]. The levels of triglyceride and total cholesterol were measured using enzymatic kits (Randox Laboratories Limited) according to the manufacturer’s instructions. The quantitative standard curve was constructed to determine triglyceride and total cholesterol levels.

### 4.7. Evaluation of Malondialdehyde (MDA) Levels in the Serum and Liver

Based on the protocol of previous study [46], the liver samples were homogenized in 1.15% KCl solution. The serum samples and liver homogenates were mixed with 2-thiobarbitutric acid and incubated in a boiled water bath for 45 min followed by cooling down. After centrifugation at 1600× *g*, 4 °C for 10 min, the samples were kept at room temperature for 30 min, and then the supernatants were collected and measured using a Luminescence Spectrometer (Hitachi, F2000, Tokyo, Japan) by excitation at 520 nm and emission at 535 nm. The quantitative standard curve was constructed to determine the level of MDA.

### 4.8. Measurement of the Activities of ACC and FAS in the Liver Tissues

Activities of ACC and FAS were measured and calculated as described previously [47]. Briefly, liver samples were homogenized in citrate buffer/tri-sodium citrate solution. After centrifugation, ACC activity was measured by mixing the supernatant with Tri-HCl buffer, magnesium chloride, monohydrate potassium citrate, glutathione, potassium hydrogen carbonate, bovine serum albumin, acetyl-CoA, and ATP, followed by NADPH, then incubating for 5–6 s. Absorbance was measured at 340 nm in 30-s intervals for 5 min using a UV–vis spectrophotometer (UV-7800, JASCO Co., Tokyo, Japan) to calculate the activity of ACC. FAS activity was measured by mixing the supernatant with potassium hydrogen phosphate trihydrate, dithiothreitol, acetyl-CoA, ethylenediaminetetraacetic acid, malonyl-CoA and NADPH, then incubating for 5–6 s. Absorbance was measured at 340 nm in 30-s intervals for 5 min using a UV-vis spectrophotometer. The quantitative standard curve was constructed to determine the levels of ACC and FAS.

### 4.9. Measurement of HSL and LPL Activity in the Abdominal Adipose Tissues

HSL activity was evaluated using methods reported in the previous study with minor modifications [48]. Briefly, isolated abdominal adipose tissues were washed with 0.9% saline, cut up, and mixed with TES buffer, followed by incubation at 37 °C and 50 rpm in a shaking incubator. After incubation for 1, 2, and 3 h, samples in Eppendorf microcentrifuge tubes were further incubated in a 70 °C water bath for 10 min followed by centrifugation. The lipolysis rate of the supernatant was measured using glycerol assay kits (Randox Laboratories Limited, Crumlin, UK) according to the manufacturer’s instructions.

LPL activity was evaluated using methods reported in the previous study with minor modifications [49]. Briefly, isolated abdominal adipose tissues were washed with 0.9% saline, cut up, and then mixed with Krebs-Ringer bicarbonate buffer, followed by incubation at 37 °C and 50 rpm in a shaking incubator. After incubation for 1 h, the samples were mixed with enzyme reaction solution and substrate solution. After incubation at 37 °C for 10 min, the incubated solution was mixed with methanol/chloroform/heptane and incubated at 42 °C for 3 min followed by centrifugation. The absorbance of the supernatant was measured at 400 nm, and the optical density value was used for the calculation of LPL activity according to the Beer–Lambert law. The quantitative standard curve was constructed to determine the levels of HSL and LPL.

### 4.10. Histopathological Examinations

Histopathological examinations of the liver tissues were conducted by oil red O staining. Deparaffinization and rehydration of tissue sections were performed by sequential immersion in xylene and ethanol. Oil red O staining was performed on 4–5-μm sections of each paraffin block of liver tissues. The tissues were excised, sectioned, and stained with oil red O by the Agricultural Technology Research Institute (Miaoli, Taiwan). The area of liver tissues was selected randomly for analysis to avoid artificial bias. The percentage of oil red O-positive area within the area of liver tissue was quantified using the ImageJ image processing and analysis program (Bethesda, MD, USA).

### 4.11. Statistical Analysis

The graphs were obtained using SigmaPlot 14.0 (Systat Software Inc.). SPSS Version 14.0 (SPSS Inc., Chicago, IL, USA) and one-way analysis of variance (ANOVA) were employed to statistically analyze the data and to determine the statistical differences between each group, respectively, with the level of significance set at *p* < 0.05. A two-tailed Student’s *t*-test was used to assess the statistical difference between the treatment group and the control group. The results are expressed as mean ± standard deviation. 

## 5. Conclusions

In this study, the process of citrus peel fermentation with *S. cerevisiae* T1 was established. After fermentation, the contents of anti-obesity components were increased. The results of animal experiments substantiate the beneficial effects of fermented citrus peel on ameliorating HFD-induced obesity. However, further studies are required to identify other anti-obesity components and to comprehensively understand the cellular and molecular mechanism of fermented citrus peel. This study clearly demonstrates the potential of fermented citrus peel to be developed into functional foods for the management of HFD-induced obesity.

## Data Availability

The datasets analyzed in the current study are available from the corresponding author upon request.
